# Supplementary material for: Molecular motor tug-of-war regulates elongasome cell wall synthesis dynamics in Bacillus subtilis
Source: Nat Commun. 2024 Jun 26;15:5411. doi: 10.1038/s41467-024-49785-x (PMC11208587; doi:10.1038/s41467-024-49785-x)
Supplement: Supplementary file 3 — Description of Additional Supplementary Files [file 41467_2024_49785_MOESM3_ESM.pdf]

Description of Additional Supplementary Files:

Supplementary Movie 1: Example of MreB singlemolecule dynamics in wild-type live B. subtilis cells imaged via smVerCINI corresponding to leftmost kymograph, Fig 1b. Scale bar, 1 μm.

Supplementary Movie 2: Example of MreB singlemolecule dynamics in wild-type live B. subtilis cells imaged via smVerCINI corresponding to rightmost kymograph, Fig 1b. Scale bar, 1 μm.

Supplementary Movie 3: Example of MreB singlemolecule dynamics in wild-type live B. subtilis cells imaged via smVerCINI. Scale bar, 1 μm.

Supplementary Movie 4: Example of MreB singlemolecule dynamics in low RodA expression level corresponding to Fig 2a. Scale bar, 1 μm.

Supplementary Movie 5: Example of MreB singlemolecule dynamics in low RodA expression level corresponding to Supplementary Figure 13, top, leftmost kymograph. Scale bar, 1 μm.

Supplementary Movie 6: Example of MreB singlemolecule dynamics in low RodA expression level corresponding to Supplementary Figure 13a, kymograph 2nd from left. Scale bar, 1 μm.

Supplementary Movie 7: Example of MreB singlemolecule dynamics in low RodA expression level corresponding to Supplementary Figure 1, top, kymograph 3rd from left. Scale bar, 1 μm.

Supplementary Movie 8: Example of MreB singlemolecule dynamics in low RodA expression level corresponding to Supplementary Figure 13, top, rightmost kymograph. Scale bar, 1 μm.

Supplementary Movie 9: Example of MreB singlemolecule dynamics in high RodA expression level corresponding to kymograph Fig 2b. Scale bar, 1 μm.

Supplementary Movie 10: Example of MreB singlemolecule dynamics in high RodA expression level corresponding to Supplementary Figure 13, bottom, leftmost kymograph. Scale bar, 1 μm.

Supplementary Movie 11: Example of MreB singlemolecule dynamics in high RodA expression level corresponding to Supplementary Figure 13, bottom, kymograph 2nd from left. Scale bar, 1 μm.

Supplementary Movie 12: Example of MreB singlemolecule dynamics in high RodA expression level corresponding to Supplementary Figure 13, bottom, kymograph 3rd from left. Scale bar, 1 μm.

Supplementary Movie 13: Example of MreB singlemolecule dynamics in high RodA expression level corresponding to Supplementary Figure 13, bottom, rightmost kymograph. Scale bar, 1 μm.
