# Supplementary material for: Molecular motor tug-of-war regulates elongasome cell wall synthesis dynamics in Bacillus subtilis
Source: Nat Commun. 2024 Jun 26;15:5411. doi: 10.1038/s41467-024-49785-x (PMC11208587; doi:10.1038/s41467-024-49785-x)
Supplement: Supplementary file 5 — Reporting Summary [file 41467_2024_49785_MOESM5_ESM.pdf]

nature portfolio

Corresponding author(s): Stuart Middlemiss, Henrik Strahl, Séamus Holden

Last updated by author(s): 30/05/2024

Reporting Summary

Nature Portfolio wishes to improve the reproducibility of the work that we publish. This form provides structure for consistency and transparency in reporting. For further information on Nature Portfolio policies, see our Editorial Policies and the Editorial Policy Checklist.

Statistics

For all statistical analyses, confirm that the following items are present in the figure legend, table legend, main text, or Methods section.

n/a	Confirmed	
☐	☒	The exact sample size (n) for each experimental group/condition, given as a discrete number and unit of measurement
☐	☒	A statement on whether measurements were taken from distinct samples or whether the same sample was measured repeatedly
☐	☒	The statistical test(s) used AND whether they are one- or two-sided<br>*Only common tests should be described solely by name; describe more complex techniques in the Methods section.*
☒	☐	A description of all covariates tested
☐	☒	A description of any assumptions or corrections, such as tests of normality and adjustment for multiple comparisons
☐	☒	A full description of the statistical parameters including central tendency (e.g. means) or other basic estimates (e.g. regression coefficient) AND variation (e.g. standard deviation) or associated estimates of uncertainty (e.g. confidence intervals)
☒	☐	For null hypothesis testing, the test statistic (e.g. F, t, r) with confidence intervals, effect sizes, degrees of freedom and P value noted<br>*Give P values as exact values whenever suitable.*
☒	☐	For Bayesian analysis, information on the choice of priors and Markov chain Monte Carlo settings
☒	☐	For hierarchical and complex designs, identification of the appropriate level for tests and full reporting of outcomes
☐	☒	Estimates of effect sizes (e.g. Cohen's d, Pearson's r), indicating how they were calculated

Our web collection on statistics for biologists contains articles on many of the points above.

Software and code

Policy information about availability of computer code

Data collection	Data collected using either Micro-Manager (v2.0gamma), NS-Elements (v5.42.02).
Data analysis	Videos were analysed using Fiji (v1.54) with open-source plugins PureDenoise-GPU and PureDenoise-CPU (https://github.com/ZikaiSun/PureGpu/tree/main).<br><br>Further data analysis done using Python (v3.9.13) and MATLAB (R2023a) with custom code available on the Holden lab Github pages:<br><br>Open source software for image analysis of VerCINI data was previously described and is available on the Holden Lab GitHub page: https://github.com/HoldenLab/VerciniAnalysisJ, https://github.com/HoldenLab/ring-fitting2,<br>Open source software for kymograph analysis available on the Holden Lab GitHub page: https://github.com/HoldenLab/Kymograph-spt-analysis.git<br>Open source PureDenoise-GPU denoising software is available on GitHub: http://www.GitHub.com/ZikaiSun/PureGpu.<br>Open source software for the tug-of-war simulations: https://github.com/HoldenLab/lipowskiModel

For manuscripts utilizing custom algorithms or software that are central to the research but not yet described in published literature, software must be made available to editors and reviewers. We strongly encourage code deposition in a community repository (e.g. GitHub). See the Nature Portfolio guidelines for submitting code & software for further information.

Data

Policy information about availability of data

All manuscripts must include a data availability statement. This statement should provide the following information, where applicable:

- Accession codes, unique identifiers, or web links for publicly available datasets
- A description of any restrictions on data availability
- For clinical datasets or third party data, please ensure that the statement adheres to our policy

Raw data are available on request to the authors and will be uploaded to a public repository prior to journal publication. Bacterial strains are available on request to the authors.

Research involving human participants, their data, or biological material

Policy information about studies with human participants or human data. See also policy information about sex, gender (identity/presentation), and sexual orientation and race, ethnicity and racism.

Reporting on sex and gender	N/A
Reporting on race, ethnicity, or other socially relevant groupings	N/A
Population characteristics	N/A
Recruitment	N/A
Ethics oversight	N/A

Note that full information on the approval of the study protocol must also be provided in the manuscript.

Field-specific reporting

Please select the one below that is the best fit for your research. If you are not sure, read the appropriate sections before making your selection.

[x] Life sciences [ ] Behavioural & social sciences [ ] Ecological, evolutionary & environmental sciences

For a reference copy of the document with all sections, see nature.com/documents/nr-reporting-summary-flat.pdf

Life sciences study design

All studies must disclose on these points even when the disclosure is negative.

Sample size

For growth curve experiments, 3 independent biological replicates were acquired per sample.
All other experiments were conducted in at least biological duplicate because variation between clonal bacterial samples was low, as estimated based on small range measured in replicate medians, unless otherwise indicated. We checked that single cell/ single molecule variability was the relevant point of statistical comparison and that variation between independent biological replicates was low based on at least two biological replicates, which is presented for all figures and analyses.

No prior sample size calculations were performed. No specific sample size was chosen (exceptions noted below) as the single cell/ single molecule nature of the measurements means moderate to large sample size, sufficient for robust statistical analysis, is usually straightforward to achieve.
For live cell single molecule experiments, as much data as possible were acquired within the 40-minute imaging window where cells have optimal physiology. This typically resulted in hundreds to thousands of datapoints.
For SIM experiments, at least 10 microscope FOVs were acquired per sample.
For cell morphology measurements, at least 100 data points were measured per sample.
For simulations, we chose to run 100 independent replicates for each condition since this gave small confidence intervals sufficient for our subsequent analyses.

Numbers of cells, track segments, and other data points are all listed in Supplementary Table 2.

Data exclusions

No data were excluded.

Replication

The number of biological replicates for each experiment, defined as the number of experiments done using independent samples, can be found in Supplementary Table 2.

Randomization

Allocating experimental groups was not relevant for this study as all bacterial cells of a particular strain are genetic clones.

Blinding

Blinding was neither possible nor necessary for this study, as 1) all bacterial cells of a particular strain are genetic clones and 2) analyses were

not sufficiently subjective to require researcher blinding.

Reporting for specific materials, systems and methods

We require information from authors about some types of materials, experimental systems and methods used in many studies. Here, indicate whether each material, system or method listed is relevant to your study. If you are not sure if a list item applies to your research, read the appropriate section before selecting a response.

Materials & experimental systems

n/a	Involved in the study
☐	☒ Antibodies
☒	☐ Eukaryotic cell lines
☒	☐ Palaeontology and archaeology
☒	☐ Animals and other organisms
☒	☐ Clinical data
☒	☐ Dual use research of concern
☒	☐ Plants

Methods

n/a	Involved in the study
☒	☐ ChIP-seq
☒	☐ Flow cytometry
☒	☐ MRI-based neuroimaging

Antibodies

Antibodies used	Anti-MreB, in-house polyclonal, originally developed by Jeff Errington lab. Glaser et al., Genes & Dev. (1997) doi:10.1101/gad.11.9.1160<br>Anti-Spo0J, in-house polyclonal, originally developed by Jeff Errington lab. Jones et al., Cell (2001), doi:10.1016/S0092-8674(01)00287-2<br>HRP-conjugated anti-rabbit IgG antibody (Sigma A6154-1ML)
Validation	Anti-MreB characterization is described in Glaser et al., Genes & Dev. (1997) doi:10.1101/gad.11.9.1160<br>Anti-Spo0J characterization is described in Jones et al., Cell (2001), doi:10.1016/S0092-8674(01)00287-2<br>Anti-Rabbit IgG Sigma A6154 product information: https://www.sigmaaldrich.com/deepweb/assets/sigmaaldrich/product/documents/649/246/a6154dat.pdf
